# Efficacy of Azadirachtin in the Integrated Management of the Root Knot Nematode *Meloidogyne incognita* on Short- and Long-Cycle Crops

**DOI:** 10.3390/plants12061362

**Published:** 2023-03-17

**Authors:** Giada d’Errico, Nicola Sasanelli, Francesco Guastamacchia, Virgilio Stillittano, Trifone D’Addabbo

**Affiliations:** 1 Department of Agricultural Sciences, University of Naples Federico II, 80055 Portici, Italy; 2 Institute for Sustainable Plant Protection—National Council or Research, 70126 Bari, Italy; 3 Sipcam Italia, 20016 Pero, Italy; 4 Experimental Zooprophylactic Institute of Latium and Tuscany “M. Aleandri”, 00178 Roma, Italy

**Keywords:** lettuce, neem, phytoparasitic nematodes, sustainable control, tomato

## Abstract

Activity of azadirachtin on phytoparasitic nematodes has been documented for some decades, but the relationship between its nematicidal efficacy and crop cycle length has not yet been clarified. This study aimed to assess the efficacy of an azadirachtin-based nematicide, for controlling the infestation of the root-knot nematode *Meloidogyne incognita*, on the short- and long-cycle crops, lettuce and tomato, respectively. Experiments on lettuce and tomato were carried out in a greenhouse infested by *M. incognita*, including non-treated soil, or treated with the nematicide fluopyram, as controls. In the experiment on the short-cycle lettuce crop, the azadirachtin product effectively suppressed *M. incognita* infestation and increased crop yield, without significant differences from fluopyram. In the tomato crop, both azadirachtin and fluopyram were not able to control nematode infestation, but resulted in significantly higher yields. Data from this study indicated that azadirachtin can be a valid alternative to fluopyram and other nematicides, for root-knot nematode control in short-cycle crops. Integration of azadirachtin with a synthetic nematicide or nematode-suppressive agronomical techniques, should be more suitable to long-cycle crops.

## 1. Introduction

The massive use of synthetic pesticides and fertilizers, required by the intensification of agricultural systems, has altered the functioning of soil systems and affected crop value and profitability, with losses of up to 20% [1]. In order to improve the sustainability of agricultural processes, the EU New Green Deal intended to restore soil biodiversity, by reducing the use of pesticides by 50%, and replacing them with more sustainable control strategies [2,3]. 

Root-knot nematodes (RKN), *Meloidogyne* spp., are among the most harmful crop pests, as damage caused by these plant parasites can be so serious as to compromise the whole crop yield, resulting in 12–15% of annual world crop losses [4,5]. In greenhouse crop systems, these losses are aggravated by the continuous crop succession, as well as by the synergistic effects deriving from the association of RKN infestation with different phytopathogenic microorganisms [6]. Therefore, soil infestation by RKN requires the adoption of adequate control strategies, mainly in the presence of high soil nematode population densities, highly susceptible crops, and favorable climatic conditions and soil textures. Control of RKN infestations has been effectively based on soil treatments with synthetic nematicides, mainly fumigants, during most of the past century [7]. The EU revision of agricultural pesticides involved most of the synthetic nematicides traditionally used for RKN management, leading to increased attention on alternative, environmentally safe, control strategies [8]. The presence of a large range of nematicidal compounds in plant species from many botanical families, has increasingly focused the attention of researchers and stakeholders to plant-based nematicidal products [9,10]. Previous studies documented a strong activity of a large number of phytochemicals, such as phenolics and glucosinolates from Asteraceae and Brassicaceae species, respectively [11,12], saponins from *Medicago* species [13], and essential oils from many aromatic and medicinal plants, on RKN [14].

The neem tree (*Azadirachta indica* Juss.), Meliaceae family, is native to southern Asia (India, Myanmar), but has also been introduced throughout tropical and subtropical regions [15]. Neem leaves, barks, seeds, and seed cakes are characterized by a large content of bioactive triterpenoid compounds [16], and their powders and extracts have been extensively documented for activity against RKN [17,18,19,20]. Chemicals obtained from neem have been documented for their toxicity to RKN since the early 1980s. In a study in 1985, Devkumar et al. [21] reported that nimbin, diacetylnimbin, and other compounds extracted from the neem seed kernel, showed nematicidal activity against the RKN *M. incognita* Kofoid et White (Chitw.).

Azadirachtin was first isolated from neem seeds in 1968 and totally synthesized in 2007 [22,23]. Among the more than 100 biologically active triterpenoid limonoids detected in neem seed kernels, azadirachtin was identified as having the most interesting potential as a pesticide, due to a favorable toxicological profile [24,25]. Azadirachtin rapidly became the active component for the development of new insecticidal products available for organic crops, due to its high activity on a number of insect pests and mites [26,27,28]. In addition, azadirachtin and its derivative products, were also investigated for their toxicity to RKN. Mojumder et al. [29] reported that a 48 h exposure to a 2000 ppm concentration of azadirachtin, resulted in more than 50% immobility of *M. incognita* second-stage juveniles (J2), also significantly decreasing their penetration into the roots of mungbean *Vigna radiata* L. seedlings. Following in vitro studies, Ambrogioni et al. [30] related this toxic effect to an ovicidal activity on RKN eggs, and to a nematostatic effect on J2. In three-year glasshouse experiments in Sicily, treatments with azadirachtin, both alone or combined with soil solarization, significantly suppressed soil population densities of *M. incognita* and gall formation on tomato roots, compared to the non-fumigant nematicides fenamiphos, ethoprophos, and fosthiazate, also providing a significant suppression of the fungal agent of tomato corky root, *Pyrenochaeta lycopersici* Cooke (Wint.) [31]. In field experiments in southern Italy, treatments with a liquid azadirachtin formulation, either alone or combined with seedling root dipping in the same product, effectively reduced the soil population density of *M. incognita* and gall formation on tomato (*Solanum lycopersicum* L.) roots, also increasing crop yield over the non-treated control [32]. Contrastingly, the laboratory assays of Lynn et al. [33] showed only a 36.3% reduction in *M. incognita* J2 motility, even after a 24 h exposure to 10 ppm of azadirachtin.

The persistence of the efficacy of azadirachtin products is related to both environmental and technical factors, such as temperature, UV, and rates and method of application, with a higher efficacy of soil treatments compared to foliar treatments [34]. Regarding the persistence of nematicidal efficacy, Javed et al. [35] reported a significant reduction in gall index and number of egg masses of *M. javanica* (Treub) Chitw., on tomato roots, even 16 weeks after soil treatments with a commercial azadirachtin product, in spite of a decline in the nematicidal performance over time. The available information is limited to long-cycle crops such as tomato; there are no data on the persistence of the nematicidal efficacy of azadirachtin on shorter-cycle crops. The aim of this study, carried out in a commercial greenhouse, was to compare and evaluate the suppressive efficacy of an azadirachtin-based product on *M. incognita* infestation, on plant growth, and on yield performance of crops with different cycle lengths, such as lettuce (*Lactuca sativa* L.) and tomato. The side effects of the azadirachtin product, on the infestation of lepidopteran insects *Spodoptera littoralis* Boisduval and *Tuta absoluta* Meyrick, were also visually monitored on both crops.

## 2. Results

Before the experiments, soil was uniformly infested with *M. incognita*, with an initial population density of 0.8 J2 mL^−1^ soil (*Pi*). At lettuce harvest, the final soil population density (*Pf*) was significantly reduced by the treatments with azadirachtin and fluopyram, compared to the non-treated control, −71% and −67%, respectively, with no significant differences between the two products (Table 1). Both treatments with azadirachtin and fluopyam also resulted in a significant reduction in gall index on lettuce roots, −47% and −51%, respectively (Table 1). 

Both the azadirachtin and fluopyram products significantly increased the lettuce yield (+36.7% and +31.1%, respectively) in comparison to the non-treated plots, again without statistical differences between azadirachtin and fluopyram (Table 1).

A uniform infestation of *M. incognita* (0.9 J2 mL^−1^ soil) was also detected before tomato transplanting (Table 2). 

**Figure 1:**
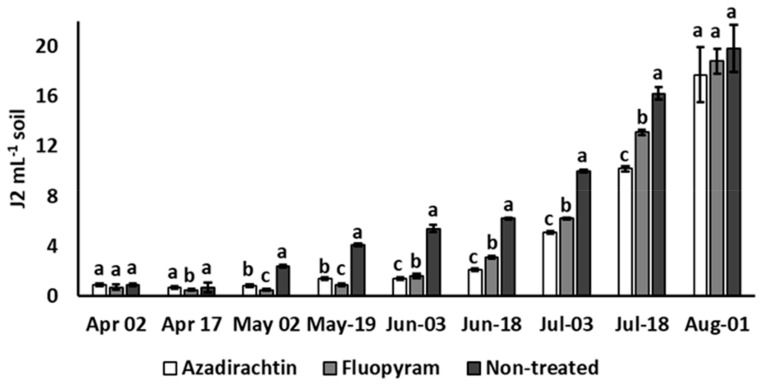
Effect of soil treatments with azadirachtin and fluopyram on soil population density of *Meloidogyne incognita* along the tomato crop cycle. Bars marked by the same letters at each sampling date are not significantly different, according to the Student–Newman–Keuls test (*p* > 0.05).

At the end of the tomato crop, the *Pf* did not differ significantly among the azadirachtin and fluopyram treatments and the non-treated control, with a *Pf* 18–23 times higher than the *Pi* (Table 2). At tomato uprooting, plant roots were heavily infested by *M. incognita*, though values of root gall index were slightly but significantly lower in soil treated with both azadirachtin and fluopyram, than in non-treated plots (Table 2). At almost all soil sampling dates throughout the experiment, the population of *M. incognita* was significantly lower in soil treated with azadirachtin or fluopyram, though consistently increasing after two months from transplanting (Figure 1). 

During the first two months, *M. incognita* population density was statistically lower in soil treated with fluopyram than in plots treated with the azadirachtin product which, conversely, resulted in significantly more suppression than fluopyram for the following 45 days of the tomato cycle (Figure 1). 

**Figure 2:**
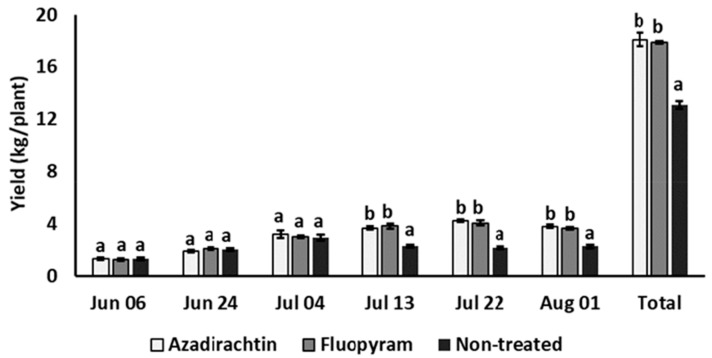
Effect of soil treatments with azadirachtin and fluopyram on tomato yield at the different harvesting dates. Bars marked by the same letters at each harvesting date are not significantly different, according to the Student–Newman–Keuls test (*p* > 0.05).

The tomato yield from the plots treated with both nematicides was significantly higher than that from the non-treated plots, +38.2% and +36.6%, respectively, without statistical differences between the two products (Table 2). No statistical differences were found among azadirachtin, fluopyram, and the non-treated control at the first three tomato harvest dates, while the yields from the plots treated with azadirachtin and fluopyram were significantly higher than from the non-treated plots at the final three harvest dates (Figure 2).

Visual monitoring of the plants along the lettuce and tomato crop cycles, indicated that the tested azadirachtin product also showed an insecticidal activity. In the lettuce experiment, plants from plots treated, or not, with fluopyram, needed three weekly treatments with a deltametrin product, starting from 25 days after the transplant, to control the African cotton leafworm *S. littoralis*, while no insect infestation occurred in the plots treated with azadirachtin. In the tomato crop, the presence of *S. littoralis* and of the tomato pinworm *Tuta absoluta*, was detected early in the cycle (29 April) in plots treated, or not, with the fluopyram product, needing four treatments with the deltametrin formulate until the end of the crop cycle. Conversely, symptoms of the two insects’ infestations appeared much later (5 June) in soil treated with the azadirachtin product, requiring only two insecticidal treatments.

## 3. Discussion

This study indicated that azadirachtin can effectively control RKN infestations on short-cycle crops, such as lettuce, providing a nematicidal performance not different from that of the synthetic nematicide fluopyram. Conversely, the azadirachtin product, at least at the doses and number of treatments applied in this study, failed to control *M. incognita* infestation until the end of a longer-cycle crop, such as tomato. In addition to the short persistence of the product, the better nematicidal performance on lettuce should also be attributed to the less conducive conditions for *M. incognita* during the period of the lettuce experiment, ending in May, than in the tomato experiment, ending in August, with a consequent stronger nematode pressure on the tomato roots. 

This study is the first report of the application of an azadirachtin product on a short-cycle crop such as lettuce, while the nematicidal activity of azadirachtin has been repeatedly documented on tomato. In a three-year experiment on a greenhouse with tomato, in Sicily, soil treatments with a commercial formulation of azadirachtin, both alone and in combination with soil solarization, were able to significantly reduce *M. incognita* infestation and increase crop yield, and protect from corky root disease caused by *P. lycopersici* [31]. Field studies in the Apulia region, southern Italy, also documented a significant reduction in the soil population of *M. incognita* and root galling on tomato, following soil treatment with an azadirachtin product, either alone or combined with an overnight root dipping of tomato seedlings with a solution of the same product [32]. The efficacy of soil treatment with azadirachtin products against *M. incognita* infestation on tomato, was further proved by other greenhouse studies in central Italy [36]. 

The effects of azadirachtin formulations on RKN infestation on tomato were contrastingly documented in small-scale experiments in pots. Javed et al. [37] reported that a 0.1% azadirachtin product significantly reduced the penetration and delayed development of *M. incognita*, within potted tomato roots, when applied at 0.05% and 0.1% w/v concentrations. In another study, a 1% azadirachtin product was able to reduce the infection rate of *M. incognita* on potted tomato, with a reduction in gall formation of up to 88%, when applied at the highest concentration tested (0.6%) [38]. A 59% reduction in gall formation on tomato roots was also reported, following soil amendments with a powder formulation of azadirachtin, in a greenhouse potted plant bioassay [39]. Beside tomato, azadirachtin products have also demonstrated their effectiveness on RKN control on other crops, such as okra (*Abelmoschus esculentus* Moench.): a reduced gall presence and an increased growth, following soil treatments with a 1,500 ppm azadirachtin formulation, both in pots and in an open field, were reported [40].

The effects of azadirachtin on RKN J2 were contrastingly reported. In the study of Ntalli et al. [41], the application of azadirachtin, either as a formulated product or as technical grade (azadirachtin 11.8%), did not affect the motility of *M. incognita* J2, except at doses > 12.8 μg a.i mL^−1^. In agreement, a 1% azadirachtin product did not demonstrate a nematostatic or nematicidal effect on *M. incognita* J2, as well as did not affect nematode hatching [37]. Contrastingly, Ambrogioni et al. [30] reported paralysis of *M. incognita* J2 after 4 days of exposure to 1.92 μg mL^−1^ of azadirachtin. In other in vitro assays with *M. incognita,* a 0.6% concentration of a 1% azadirachtin product, increased J2 mortality and reduced egg hatching by 68% and 85%, respectively, compared to the water control [38]. In addition to azadirachtin concentration and type of adjuvant used for preparing test solutions, this variability was also related to the solvents used in the formulation [42], as well as to the pH of the solutions [43]. 

The mode of action of azadirachtin on RKN has still not been elucidated, but the shared physiological roles suggest mechanisms similar to those widely investigated on insects. The modes of action of azadirachtin on insects, as reviewed by Murdue [44], include an alteration of insect feeding and growth, as well as abnormal and delayed molts and sterility, due to the direct action of azadirachtin on cells and neuroendocrine tissues. In the previous study of Rembold et al. [45], interference of azadirachtin with molting hormone pools, was suggested as the main mechanism of the strong effect of this compound on the molts of insect species from different orders, such as the Mexican bean beetle (*Epilachna varivestis* Muls.), the Mediterranean flour moth *Ephestia kuehniella* Zell., and the honeybee, *Apis mellifera* L. 

The significant yield increase obtained from both lettuce and tomato crops, is in agreement with data from all the previous applications of azadirachtin products in field and greenhouse experiments [31,33,36,40]. In the absence of studies on specific effects of azadirachtin on plants, the increased yield may be reasonably attributed to the improved plant nutrition, deriving from the reduced nematode pressure on the root system [46].

The complete control, or the delayed insurgence, of the insect *S. littoralis*, observed on lettuce and tomato crops, respectively, was in full agreement with literature studies. Martinez and Van Emden [47] described a number of effects, such as growth rate reduction, molting disruption, morphological anomalies, and even mortality, on *S. littoralis* larve fed on an artificial diet treated with azadirachtin. Similarly, a study of Gelbič and Němec [48] reported a significant increase in body mass, associated with precocious transformation to the pupal stage, as well as a restriction of reproduction, following the treatment of *S. littoralis* with azadirachtin or neem oil. Literature data also confirmed the contribution of azadirachtin to the control of *T. absoluta* on tomato crop. Jallow et al. [49] reported a significant reduction in *T. absoluta* infestation, and on number of damaged tomato fruits in a greenhouse, following combined treatments with azadirachtin and the biocontrol agents *Bacillus thuringiensis* Berliner or *Beauveria bassiana* (Bals-Criv.) Vuill. A further added value of azadirachtin is represented by its possible application in combination with entomopathogenic and slug-parasitic nematodes, largely used in organic and integrated crop systems, due to the stated safety of azadirachtin for these biocontrol agents [50].

This study also confirmed the nematicidal effectiveness of fluopyram against *M. incognita* on tomato, in agreement with previous literature reports [51,52]. The combination of azadirachtin with fluopyram or other synthetic nematicides, could be an effective response to the limited-in-time activity of azadirachtin treatments in long-cycle crops. In these conditions, chemical treatments should be applied before or immediately after crop transplant, to strongly reduce the initial nematode population. In organic systems, synthetic nematicides could be replaced by non-chemical control tools, such as soil solarization, biofumigation, or cover crops [53,54].

## 4. Materials and Methods 

The lettuce and tomato crops were carried out in parallel, in a cold greenhouse, located at Vitulazio (province of Caserta, southern Italy), with a slightly basic (pH 7.2) soil of medium texture. The soil was uniformly infested by the RKN *M. incognita* (0.8 J2 mL^−1^ soil, from soil samples collected within a 25 cm depth), with an average of 8.2 gall index on the roots of the previous tomato crop (21 December 2020). The nematode species was first identified at morphological level, according to the observation of female perineal patterns and measurements of diagnostic characters of adults and J2 [55]. The identification was then confirmed at molecular level, by extracting genomic DNA from individual nematodes and amplifying the crude DNA by using the SCAR primer sets Finc/Rinc and INCK14F/INCK14 [56]. The *Pi* of *M. incognita* was determined by extracting the nematode J2 from composite 500 mL soil samples, with the nematode cotton wool filter method [57], while the root gall index was estimated according to the 0–10 scale of Zeck [58]. 

The two experimental areas were uniformly rotavated and then subdivided into 12 plots of 38.4 m^2^ (8 × 4.8 m), aligned in parallel to the longer side of the greenhouse and arranged according to a randomized block design.

Lettuce cv. Manita and tomato cv. Gotico F1 were transplanted on 12 April 2021. Six-leaf lettuce seedlings and four-leaf tomato seedlings were transplanted in single rows, at a density of 384 (0.40 m apart in the row and 0.25 m between rows) and 104 (0.30 m apart in the row and 1.20 m between rows) plants per plot, respectively. 

A commercial, emulsifiable azadirachtin formulation (Oikos^®^, Sipcam, Pero, MI, Italy), containing 2.4% azadirachtin A, was applied at a dose of 1.5 L ha^−1^ at transplant, repeating the treatment three or four times at 12-day intervals, on lettuce and tomato, respectively. The product was distributed by water drip irrigation, in a 6000 L ha^−1^ volume of water acidified with phosphoric anhydride, to avoid a rapid decomposition of azadirachtin by a high soil pH, preceded and followed by an irrigation with water volumes of 15,000 and 9000 L ha^−1^, respectively. Non-treated plots, and plots treated with a commercial formulation of the synthetic nematicide fluopyram (Velum Prime^®^, Bayer CropSciences Italia, Milano, Italy, a.i. 400 g L^−1^ fluopyram), were included as controls. In both experiments, the fluopyram formulate was applied twice, at a 0.625 L ha^−1^ dose, 2 days before and 15 days after transplanting. As for the azadirachtin product, the treatment was applied as described before.

The plants of both lettuce and tomato were visually inspected at weekly intervals, to detect the presence of the insects *Spodoptera littoralis* and *Tuta absoluta*. 

The lettuce and tomato experiments were carried out for 45 and 120 days, respectively. At the end of the lettuce crop, the weight of heads was recorded in each plot. Gall index was estimated on all plants from each plot, according to Zeck’s scale [58]. In the tomato experiment, fruits were harvested six times on all plants of each plot, and the root gall index was estimated at harvest. The *Pf* of *M. incognita* was determined at the end of the lettuce crop, while it was monitored at 12-day intervals from transplanting to final harvest of the tomato crop. Nematodes J2, either already present in the soil or emerged from the nematode eggs, were extracted from 10-subsample composite soil samples collected from each plot, using the nematode cotton wool filter method [56], and counted under a stereoscopic microscope.

All data were statistically analyzed by the analysis of variance (ANOVA), comparing means by the Student–Newman–Keuls test (*p* ≤ 0.05). Statistical analyses were performed by using the software PlotIT 3.2 (Scientific Programming Enterprises, Haslett, MI, USA).

## 5. Conclusions

The results from this study are a further confirmation that azadirachtin and its derivatives can be an effective tool for the management of RKN on horticultural crops, providing nematicidal performances even comparable to those of the synthetic nematicide fluopyram. Use of azadirachtin-based nematicides could be particularly suited to organic crop systems, due to the poor availability of effective nematicidal control tools, though it could also be easily included in integrated nematode management strategies. Data from the experiment on lettuce, indicated that azadirachtin products should be preferred to fluopyram or other synthetic nematicides, as it also ensured an effective control of insect pests without additional costs. However, the fungicidal properties of fluopyram, suggest the combined use of both products, at half dosage, in the presence of soil-borne fungal phytopathogens [59]. Concerning the experiment on tomato, that is a long-cycle crop, azadirachtin is not able to ensure full crop protection from RKN throughout the whole cycle. In these crops, an integration with synthetic nematicides or non-chemical control techniques could effectively improve the efficacy of treatments with azadirachtin products.

## Figures and Tables

**Table 1:** Effect of treatments with azadirachtin and fluopyram on the control of *Meloidogyne incognita* on lettuce and on crop yield, 45 days after transplant.

Treatment	Yield (kg/plot)		*M. incognita* (J2 mL^−1^ soil)				Gall Index (0–10)	
			Pi ^3^		Pf ^4^			
Azadirachtin	112.9 ± 3.4 ^1^	b ^2^	0.8 ± 0.1	a	1.5 ± 0.1	c	3.9 ± 0.2	b
Fluopyram	108.3 ± 3.3	b	0.9 ± 0.1	a	1.7 ± 0.1	b	3.6 ± 0.2	b
Non-treated	82.6 ± 3.4	a	0.8 ± 0.1	a	5.1 ± 0.1	a	7.3 ± 0.6	a

^1^ Each value is a mean of four plots, each plot with 384 plants (mean ± standard deviation). ^2^ Means followed by the same letters on the same column are not significantly different, according to the Student–Newman–Keuls test (*p* > 0.05). ^3^ *Pi*, initial nematode population density. ^4^ *Pf*, final nematode population density.

**Table 2:** Effect of treatments with azadirachtin and fluopyram on the control of *Meloidogyne incognita* on tomato and on crop yield, 120 days after transplant.

Treatment	Yield (kg/plot)		*M. incognita* (J2 mL^−1^ Soil)				Gall Index (0–10)	
			Pi ^3^		Pf ^4^			
Azadirachtin	18.1 ± 0.5 ^1^	b ^2^	0.9 ± 0.1	a	17.8 ± 2.2	a	7.4 ± 0.6	b
Fluopyram	17.9 ± 0.1	b	0.8 ± 0.2	a	18.8 ± 1.0	a	7.2 ± 0.5	b
Non-treated	13.1 ± 0.3	a	0.9 ± 0.1	a	19.8 ± 1.9	a	8.8 ± 0.6	a

^1^ Each value is a mean of four plots, each plot with 104 plants (average ± standard deviation). ^2^ Means followed by the same letters on the same column are not significantly different, according to the Student–Newman–Keuls test (*p* > 0.05). ^3^ *Pi*, initial nematode population density. ^4^ *Pf*, final nematode population density.

## Data Availability

All data is available upon request from the corresponding author.
